# Visualizing tropoelastin in a long-term human elastic fibre cell culture model

**DOI:** 10.1038/srep20378

**Published:** 2016-02-04

**Authors:** M. Halm, K. Schenke-Layland, S. Jaspers, H. Wenck, F. Fischer

**Affiliations:** 1 Research and Development, Beiersdorf AG, Unnastr. 48, Hamburg, Germany; 2 Dept. of Women’s Health, Research Institute for Women’s Health, Eberhard-Karls-University Tübingen, Silcherstr. 7/1, 72076 Tübingen, Germany; 3 Dept. of Cell and Tissue Engineering, Fraunhofer Institute for Interfacial Engineering and Biotechnology (IGB), Nobelstr. 12, 70569 Stuttgart, Germany; 4 Dept. of Medicine/Cardiology, Cardiovascular Research Laboratories, David Geffen School of Medicine at UCLA, 675 Charles E. Young Drive South, MRL 3645, Los Angeles, CA, USA

## Abstract

Elastin is an essential protein found in a variety of tissues where resilience and flexibility are needed, such as the skin and the heart. When aiming to engineer suitable implants, elastic fibres are needed to allow adequate tissue renewal. However, the visualization of human elastogenesis remains in the dark. To date, the visualization of human tropoelastin (TE) production in a human cell context and its fibre assembly under live cell conditions has not been achieved. Here, we present a long-term cell culture model of human dermal fibroblasts expressing fluorescence-labelled human TE. We employed a lentiviral system to stably overexpress Citrine-labelled TE to build a fluorescent fibre network. Using immunofluorescence, we confirmed the functionality of the Citrine-tagged TE. Furthermore, we visualized the fibre assembly over the course of several days using confocal microscopy. Applying super resolution microscopy, we were able to investigate the inner structure of the elastin–fibrillin-1 fibre network. Future investigations will allow the tracking of TE produced under various conditions. In tissue engineering applications the fluorescent fibre network can be visualized under various conditions or it serves as a tool for investigating fibre degradation processes in disease-in-a-dish-models.

Elastic fibres are the main component of the extracellular matrix (ECM) in connective tissues like blood vessels, lung and skin. They provide elastic recoil and elasticity to these tissues^1^. The importance of elastic fibres and elasticity of certain tissues is underlined by a group of hereditary disorders where mutations in genes of elastic fibre components lead to severe phenotypes like Cutis Laxa or Marfan syndrome^1^. The importance of elastin and elastic fibres for a healthy tissue physiology makes them a crucial component for tissue engineering aiming to replace or repair destroyed or injured elastic tissues^2^,^3^, like the skin, suffering from full-thickness skin wounds^4^, or defective heart valves^5^.

The complex structure of elastic fibres consists of a diverse set of molecules with elastin being the dominant component of mature elastic fibres. The soluble monomer of elastin is tropoelastin (TE), which is secreted from elastogenic cell types, mainly from fibroblasts and vascular smooth muscle cells (VSMCs). TE is an at least 60 kDa large mature protein, depending on splice variants, and is thought to be chaperoned to the cell surface by the elastin binding protein (EBP)^6^, where it self-assembles (=coacervates) into globular structures. In the ECM, microfibrils, mainly consisting of fibrillin-1, serve as a scaffold where TE globules are deposited^7^ and subsequently cross-linked via enzymes of the lysyl oxidase (LOX) family^8^. TE gene expression is highest during foetal and early neonatal development, with little turnover of the mature elastic fiber^9^. Although mature elastic fibres are mainly synthesized during early development^10^, elastic fibre generation can be triggered as a part of wound healing in adults. However, adult tissues lose the ability to generate correctly assembled new fibres or to repair old fibres^11^. Furthermore, the resulting fibres are of poorer quality compared to those formed during development^12^.

We previously developed and characterized an *in vitro* cell culture model using neonatal human dermal fibroblasts (HDFneo) stimulated with transforming growth factor beta 1 (TGF-β1)^13^ for a better understanding of elastogenesis. It was demonstrated that TGF-β1 is a potent inductor for the generation of mature elastic fibres. In detail, high-performance liquid chromatography (HPLC) revealed a higher desmosine/isodesmosine content, multiphoton laser scanning microscopy (MPLSM) measurements showed an elastin-specific autofluorescence that is typical for mature fibres, and quantitative real time – PCR (qRT-PCR) demonstrated an induction of the genes LOX und lysyl oxidase homolog 1 (LOXL1)^13^. In this previous model, visualization of TE/elastin was done via antibody labelling in fixed cell culture models. However, this method lacks the option of dynamic or long-term visualization. Looking for alternatives, we found the fluorescence labelling of TE via a fluorescence tag is a useful tool. This has be done in different models, e.g. via transient expression of a bovine TE-timer construct in RFL-6 cells (rat lung fibroblasts)^7^ or via stable expression of rat-TE in rats or rat VSMCs using an adenoviral system^14^.

To our knowledge, a model to express labelled human TE in human skin cells has not been described. We transduce HDFneo with lentivirus carrying a Citrine-TE construct to overexpress labelled TE. We show that this overexpression of fluorescence-labelled TE is stable over multiple passages and able to generate fluorescent elastic fibres in a culture period of 14 days as confirmed via immunofluorescence analysis (IFA) and confocal microscopy. Using live cell imaging, we were able to visualize dynamics in elastogenesis. We also show that Citrine is a suitable marker for ground state depletion (GSD) microscopy to further investigate the fine structure of these *in vitro* generated fibres. This fluorescence *in vitro* model allows the study of the dynamics of elastic fibre formation in the skin. Its fluorescent fibres might serve as a useful tool to study tissue physiology.

## Results

To add the option of a dynamic or long-term visualization of elastogenesis to our previously established elastic fibre model^13^, we transfected HDFneo with a plasmid carrying an EGFP-labelled TE. However, none of the different transfection methods chosen (lipofection, electroporation, see Supplementary Fig. S1) was suitable to successfully generate EGFP-TE expressing cells. Therefore, we chose viral transduction as a biological method to overexpress TE in HDFneo, which also bears the advantage of a stable expression of fluorescent TE, in contrast to a transient expression via physical transfection methods. Additionally, we changed the fluorophore to Citrine as Citrine had been described to be suitable for high-resolution light microscopy^15^.

### qRT-PCR shows overexpression of Citrine-elastin in transduced HDFneo while fibrillin-1 expression is not altered

To assess the level of overexpression of elastin mRNA in transduced HDFneo, relative gene expression levels were determined via qRT-PCR in transduced and non-transduced HDFneo, both additionally stimulated with TGF-β1 as a potent stimulator for elastic fibre formation *in vitro*^13^. Furthermore, mRNA levels of the fluorescent tag Citrine and of the elastic fibre component fibrillin-1 were measured.

**Figure 1:**
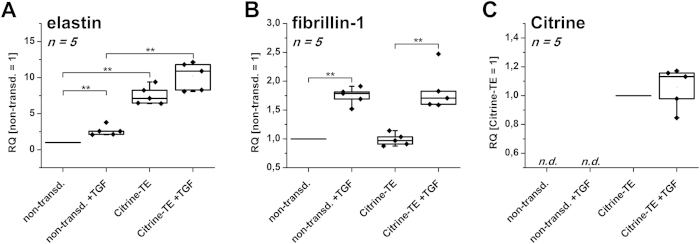
Gene Expression Analysis of non-transduced and Citrine-TE transduced HDFneo. Relative gene expression of elastin (**A**), fibrillin-1 (**B**) and Citrine (**C**) in non-transduced (non-transd.) and transduced (Citrine-TE) HDFneo, treated for 24 h with 10 ng/mL TGF-β1 (TGF) or left untreated as a control. Expression levels were normalized to 18 S rRNA as housekeeping gene and are given as fold differences relative to non-transduced (or Citrine-TE respectively) samples without TGF-β1. n = 5. **p < 0.01 Mann-Whitney test.

Figure 1 depicts the relative mRNA expression of elastin, fibrillin-1 and Citrine as median relative fold change of five independent experiments (averages and standard deviations are found online as Supplementary Table S1). Fold changes were calculated from the normalized expression levels using the untreated non-transduced (or the transduced respectively) cells as reference.

In non-transduced cells, 24 h incubation with TGF-β1 led to a 2.56-fold induction of elastin mRNA levels when compared to untreated cells (Fig. 1A). In comparison to non-transduced cells, Citrine-TE transduced HDFneo showed a significant 7.12-fold increase in elastin expression compared to the untreated cells. A 10.9-fold increase was observed when transduced cells were additionally incubated with TGF-β1, which is significantly higher than the mRNA levels of non-transduced TGF-β1-treated cells. But, there was no significant change relative to the transduced untreated cells. Fibrillin-1 mRNA levels were not significantly changed in transduced cells compared to the non-transduced cells, although TGF-β1-treatment significantly induced fibrillin-1 in both experiments (1.79-fold and 1.71-fold, respectively). The probe for Citrine did not detect any correspondent mRNA in the non-transduced cells. In the Citrine–TE transduced cells, the Citrine mRNA was well detectable. Citrine levels did not change with TGF-β1 treatment.

### IFAs reveal that Citrine-elastin is incorporated into the elastic fibre network

**Figure 2:**
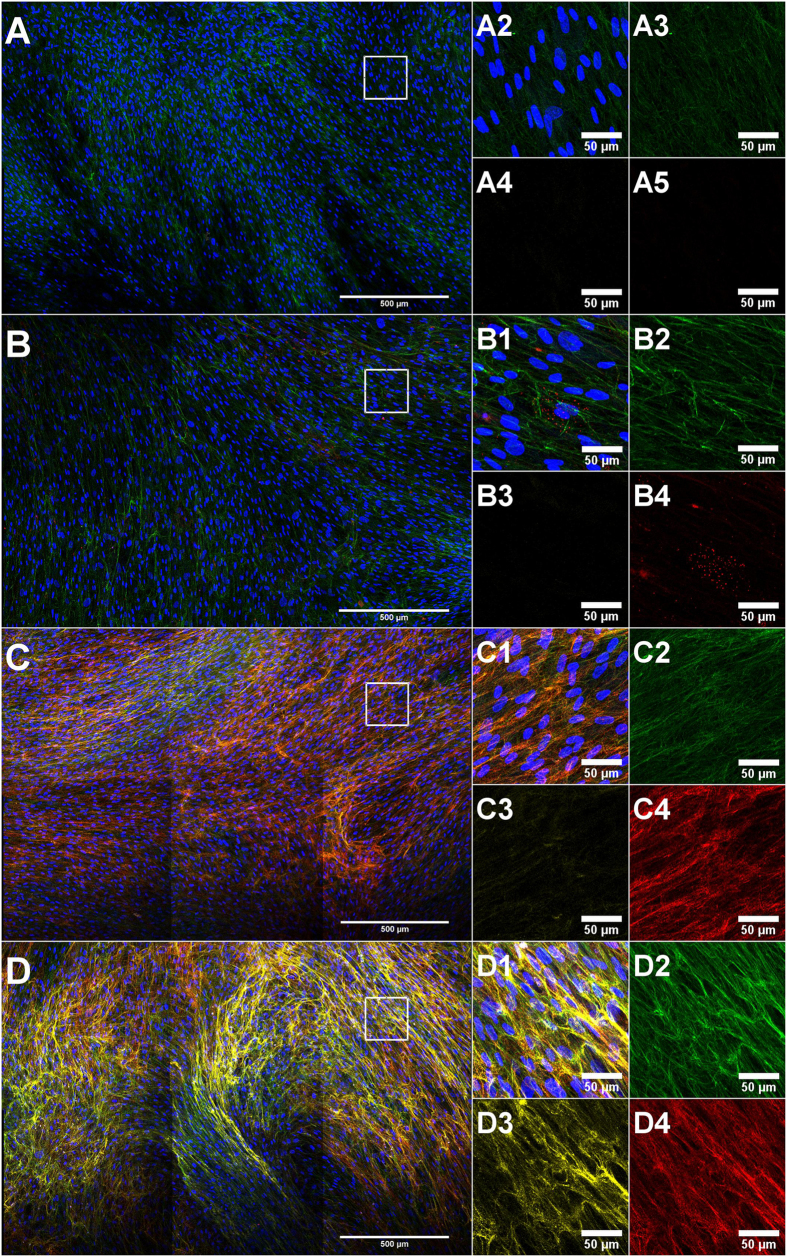
Microscopic evaluation of fluorescence-labelled TE overexpressed in an *in vitro* cell culture model. Non-transduced (**A**,**B**) and Citrine-TE transduced (**C**,**D**) HDFneo were cultured with 10 ng/mL TGF-β1 (**B**,**D**) or left untreated (**A**,**C**) as a control. Immunostaining with DAPI (blue), anti-human fibrillin-1 (green) antibody and anti-human TE/elastin (red) antibody. The fluorescent protein Citrine is shown in yellow. Depicted are representative mosaic images as maximum projections, acquired with a 20x dry objective on a Leica TCS SP5 microscope. Large images: stitched 2 × 3 mosaic of merged single channels. Small images: zoomed detail (white square) of the corresponding large image as merged (**1**) and single channel images (**2** fibrillin-1; **3** Citrine; **4** TE/elastin). Representative images of n = 3.

In order to investigate whether the fluorescence-labelled TE expressed in HDFneo is stably expressed and functional in terms of the ability to generate elastic fibres, transduced and non-transduced HDFneo were cultured with and without the presence of TGF-β1 for a period of 14 days, followed by fixation and labelling with antibodies against TE/elastin and fibrillin-1. Secondary antibodies were chosen in a way that a separate acquisition of Citrine, TE/elastin and fibrillin-1 was possible. Figure 2 depicts representative images as maximum projections of three independent experiments acquired with the confocal laser scanning microscope (CLSM) as 2 × 3 mosaics (see methods for further details). A network of fibrillin-1 microfibrils was visible after 14 days in all four different conditions with no obvious differences in fibrillin-1 deposition with respect to Citrine–TE transduction or TGF-β1 treatment (Fig. 2A–D, green: fibrillin-1). TE/elastin antibody staining (red in Fig. 2) was not detectable in the untreated non-transduced fibroblasts (Fig. 2A), and only a weak staining of elastic fibres was observed in the corresponding TGF-β1-treated cultures. In contrast, the transduced fibroblasts showed a strong staining for TE/elastin. Both the TGF-β1-treated and the untreated sample (Fig. 2C,D) appeared similar in terms of deposited amounts of TE/elastin and organization of the elastic fibre network. A Citrine signal (yellow in Fig. 2) was only apparent in the transduced fibroblasts (Fig. 2C,D). The signal was stronger in the TGF-β1 treated sample. Under both conditions, Citrine showed a fibrillar organized structure and was co-localised with the elastic fibre network.

### Quantification of deposited fibre components

**Figure 3:**
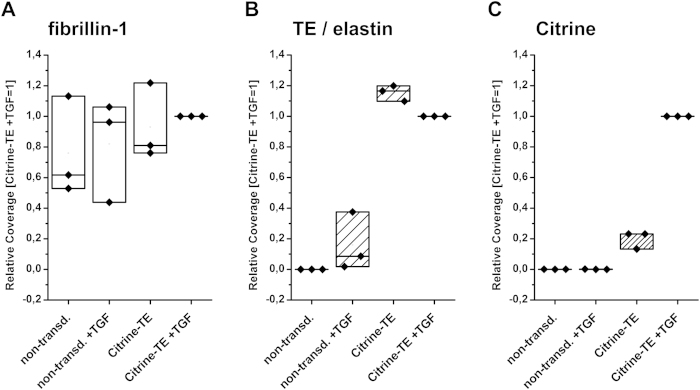
Image analysis of immunostained cell culture models. Image analysis of cell culture models of non-transduced (non-transd.) and Citrine-TE transduced HDFneo, based on the single channel images shown in Fig. 2. Using an ImageJ based algorithm, the relative signal coverage of the channels representing fibrillin-1, TE/elastin and Citrine were determined and normalized to the values for the TGF-β1 (TGF) stimulated Citrine-TE transduced cells within each of three experimental set-ups. The values obtained for the analysed proteins are shown as Box Plots for fibrillin-1 (**A**), TE/elastin (**B**) and Citrine (**C**). n = 3. Further details: see image analysis in methods. Using the Mann-Whitney-Test there were no significant differences detectable.

To quantify differences in the amount of deposited fibrillin-1, TE/elastin and Citrine-TE between the distinct conditions of this *in vitro* cell culture model, we used an ImageJ based software to determine the values for signal coverage per single image of a mosaic (details, see image analysis in methods). The results obtained from three experimental set-ups using this evaluation method, in which the values for TGF-β1-treated Citrine-TE transduced cells were set to 100% within each set-up, are provided in Fig. 3 (averages and standard deviations are found online as Supplementary Table S2). The relative signal coverage for fibrillin-1 (Fig. 3A) fluctuated over a wide range, but appeared to be independent of TGF-β1 stimulation or transduction with Citrine-TE. However, there was a trend towards lower coverage in the TGF-β1 unstimulated samples (median values: non-transduced 0.62; non-transduced + TGF 0.96; Citrine-TE 0.81; Citrine-TE + TGF 1.00). The signal coverage for TE/elastin differed greatly between transduced and non-transduced HDFneo (Fig. 3B). Non-transduced cells showed in median a relative coverage of 0 when untreated and 0.09 when treated with TGF-β1. Citrine-TE transduced cells reached a much higher coverage for TE/elastin, which was highest when cells were left untreated (1.17 in median compared to 1 for TGF-β1 treated cells). As depicted in Fig. 2, the relative signal coverage for Citrine in non-transduced cells was 0 for both TGF-β1-stimulated and untreated cells. For transduced cells, not treated with TGF-β1, a median coverage of 0.23 could be determined compared to 1.00 for TGF-β1-treated cells (see Fig. 2D compared to Fig. 2C).

### Ground state depletion (GSD) microscopy reveals ultrastructure of elastic fibres

**Figure 4:**
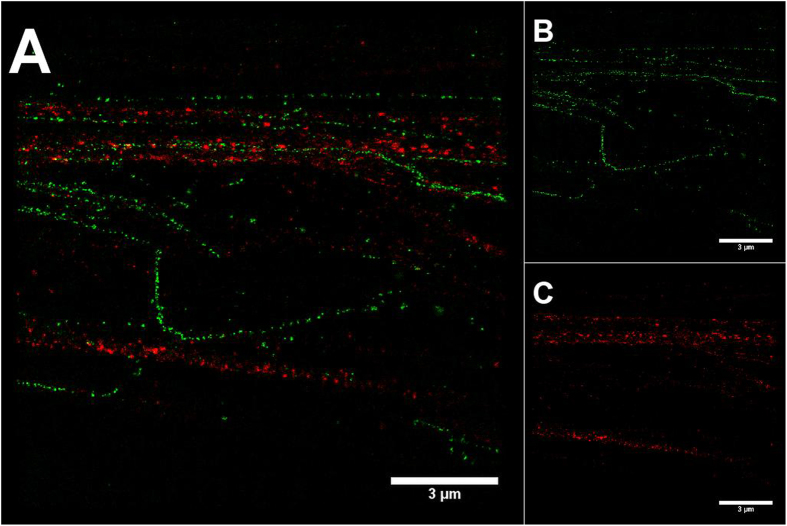
GSD images of unfixed and immunostained HDFneo expressing Citrine-TE. Transduced HDFneo were stimulated for 7 d with 10 ng/mL TGF-β1, immunostained unfixed for fibrillin-1 and imaged in GluOx/MEA-buffer with the Leica SR GSD using a 100x immersion objective. Antibody staining for fibrillin-1 is shown in green, Citrine fluorescence in red. Camera frame rate: 200 Hz (**B**), 100 Hz (**C**). Laser intensity and wavelength: 16.6 kW/cm^2^ and 642 nm (**B**), 5.2 kW/cm^2^ and 488 nm (**C**). Number of camera frames: 20.000 (**B**), 16.329 (**C**). **A** Merged image. Representative image of n = 3.

**Figure 5:**
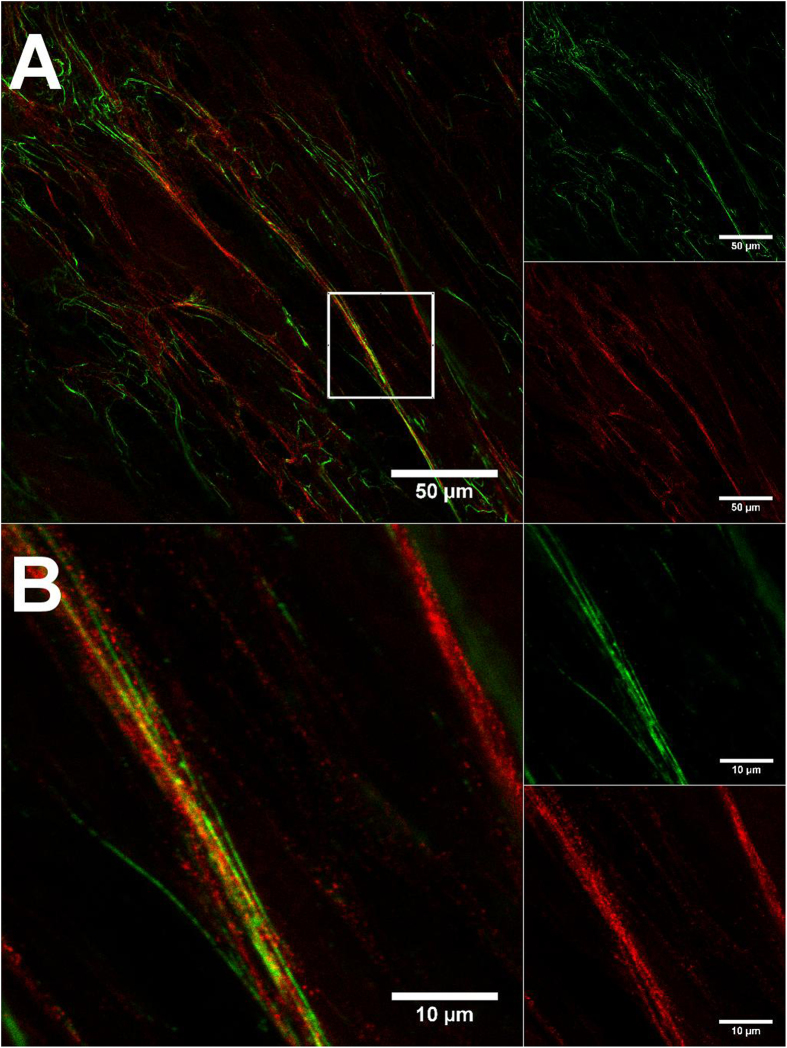
Fluorescence images of unfixed and immunostained HDFneo expressing Citrine-TE. Transduced HDFneo were stimulated for 12 d with 10 ng/mL TGF-β1, immunostained unfixed for fibrillin-1 and imaged in anti-bleaching DMEM with the Leica TSC SP5 using a 63x immersion objective. Antibody staining for fibrillin-1 is shown in green, Citrine fluorescence in red. (**A**) 1x zoom. (**B)** 5x zoom of the same position marked in (**A)**. Representative image of n = 3.

We used GSD microscopy in comparison to confocal microscopy to investigate the ultrastructure of the fluorescent elastic fibres. The cells were cultured for 7d with TGF-β1 and immunostained for fibrillin-1. Image acquisition for GSD microscopy was carried out as described in the methods section. Figure 4 shows a representative GSD image of Citrine-TE and fibrillin-1 fibres. There were only a few co-localised signals present, and in contrast to CLSM images (Fig. 5), both the Citrine-TE and fibrillin-1 signals appeared beaded, with the fibrillin-1 beads smaller and closer together and the Citrine-TE-beads bigger and further apart. Most of the Citrine beads seemed to border the beaded fibrillin-1 as they appeared mostly in close proximity to the fibrillin-1 fibre.

### Formation of the elastic fibre network can be observed using live cell imaging

**Figure 6:**
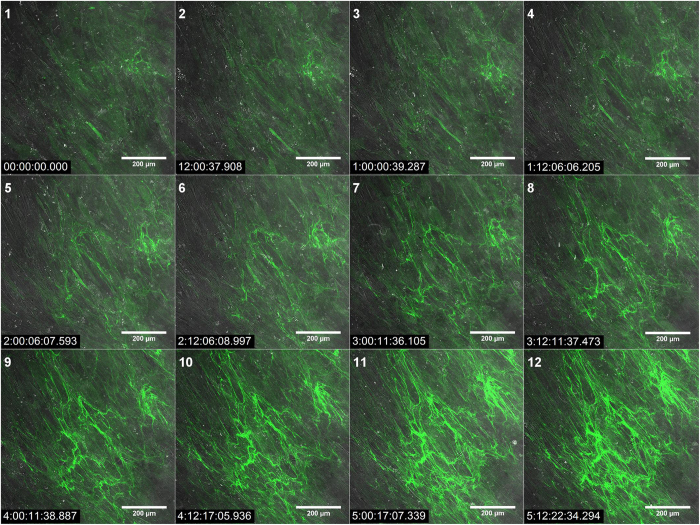
CLSM images of a growing fluorescing fibre network (day 7–14) of Citrine-TE transduced HDFneo. HDFneo were grown in chambered coverglasses and stimulated for 7 d with 10 ng/mL TGF-β1 prior to the start of imaging. On day 7 the medium was changed to TGF-β1 (10 ng/mL) containing DMEMgfp-2 and live cell imaging was carried on a Leica TSC SP5 using a 20x dry objective as described in methods for another 7 days. Shown here are images of every twelfth hour as maximum projections of the z-stacks with merged Citrine (green) and transmission (grey) images. Relative timestamp: dd:hh:mm:ss.

We imaged Citrine-TE transduced HDFneo (stimulated with TGF-β1) every hour over a period of 7 days on a confocal microscope. Images from every twelfth hour (until day 5.5) are displayed in Fig. 6 (a corresponding video is available in the Supplementary Information). As the cells were pre-stimulated with TGF-β1, there were a few fine fibres visible at the start of image acquisition. These thin fibres got thicker with time, while new fibres appeared. We used the method described in “image analysis” to determine the amount of deposited Citrine over time (see Supplementary Fig. S2). Plotting the received relative signal coverage for Citrine per image against the acquisition time resulted in a growth curve of the developing Citrine-TE network. Within the first 48 h the fluorescence rose from 9.2% to 19.6% coverage per image and was 34.4% after 96 h. Within the next 48 h the fluorescence signal coverage nearly doubled to 62.3% and seemed to reach a plateau of about 72% at the end of the acquisition. After more than 5 days, a clear and strong network of fluorescent fibres can be observed. In addition, the video shows that fibroblasts are moving along the fibre direction. Interestingly, the fibres themselves are not stiff but are rather stretched or bended in the course of acquisition time.

## Discussion

The complex network of elastic fibres is an essential element of the ECM of the dermis of the skin, heart valves or blood vessels. Although elastin and the formation of elastic fibres have been broadly studied, there are still little insights into the biology and the dynamics of fibre formation in the human skin. Our lab previously established an *in vitro* cell culture model using human dermal fibroblasts that were stimulated with TGF-β1 in order to generate an *in vitro* fibre network showing characteristics described for *in vivo* fibers^13^. Using only immunofluorescence analysis, this model lacks the opportunity to visualize growing fibres or the dynamics of fibre assembly. One way to study elastic fibre formation online and live is a fluorescent tag. So far, there are only a few models expressing fluorescence-labelled TE, but none uses a human TE construct in human skin cells. Kozel *et al*.^7^ used a bovine TE-Timer construct, which was transiently expressed in rat lung fibroblasts showing that elastic fibre formation is a multistep process via a microassembly on the cell surface and a macroassembly and deposition into the extracellular space. But, as the fluorescence-labelled TE is only transiently expressed the formation of a whole fibrous network could not be shown. Xiong *et al*.^14^ demonstrated in an abdominal aortic aneurism model in rats that the overexpression of a GFP-labelled recombinant rat elastin had a positive impact on the adverse aortic diameter alterations after perfusion. In a model of myocardial infarction in adult rats, the injection of rat bone marrow stromal cells (BMSCs) overexpressing human elastin led to significant functional improvement compared to the controls^16^. Although these experiments underline the importance of elastin for restoring healthy tissue physiology, there is currently no model using human TE in a human cell environment, which allows a dynamic study of elastin and its fibre assembly.

We therefore aimed for a long-term elastic fibre model. We chose a human TE sequence and Citrine as a fluorescence tag to be overexpressed in HDFneo. As fibre formation takes place in a time course of several days, we chose to express the tag via a lentiviral system to achieve a stable rather than a transient expression. qRT-PCR revealed that even after two passages, overexpression of TE was well detectable in transduced HDFneo (Fig. 1A). TE-overexpression was (at least after 24 h with exposure to TGF-β1) even stronger than induction of TE via stimulation with TGF-β1 in non-transduced cells. As the vector containing the Citrine-TE construct carries a cytomegalovirus (CMV) promotor, Citrine-TE was constitutively expressed and was not subjected to external regulation. Although TGF-β1 stimulation further enhanced TE levels, this induction was not significant and can be attributed to the endogenous elastin expression. Expression levels of fibrillin-1, as an example for another main fibre component, were not altered with the overexpression of elastin (Fig. 1B). However, TGF-β1 also increased fibrillin-1 levels in both transduced and control cells, underlining that despite the overexpression of elastin, the cell’s endogenous response to this stimulus was not altered. As expected, Citrine mRNA was only detectable in the transduced cells (Fig. 1C) and was not altered by TGF-β1, since the CMV promotor does not react to an exogenous stimulus.

In order to study elastic fibre formation via visualization of a fluorescence-labelled TE, it is most important that the labelled TE is functional by means of ability to generate fibres. The immunofluorescence images (Fig. 2C,D) after 14 days of cultured transduced HDFneo clearly showed that Citrine (and thus Citrine-TE) built up a fibrous network, which was co-localised to the elastic fibre network detected via antibody staining. This indicates that the human elastin sequence chosen for overexpression is functional in our cell culture model. Interestingly, the overexpression of Citrine-TE alone was sufficient to generate an elastic fibre network without the need for TGF-β1 stimulation (Fig. 2C), and the resulting fluorescence signal was about 10-times stronger (Fig. 3B) than it was achieved with TGF-β1 stimulation in the non-transduced cells. Although TGF-β1 increased fibrillin-1 mRNA levels, the resulting microfibrillar network was not impacted (Fig. 2A–D2 and 3A). In contrast, TGF-β1 did not increase Citrine mRNA levels but led to an about 5-times higher deposition of Citrine-TE without changes in overall TE/elastin-deposition. This indicates that overexpression of elastin alone might lead to a fibrillar network, but that TGF-β1 might stimulate further components of elastogenesis (apart from fibrillin-1) that facilitate elastin deposition. In this case, one would expect a stronger TE/elastin-network via antibody detection. On the other hand, it could be that the antibody detection is already at its maximum capacity so that incorporation of further elastin into to the fibrous network does not cause any change in the relative signal coverage.

Using live cell and super resolution microscopy, we further wanted to explore our model of Citrine-labelled TE. In unfixed cells (Fig. 5), we identified a colocalisation of fibrillin-1 with Citrine fibres. Magnifying structures of the microfibrillar network that were strongly loaded with Citrine (Fig. 5B) revealed that the fine structure of the fibres resembled more a fibrillin-1 fibre covered with Citrine-TE globules, rather than being an amorphous elastin core surrounded by an outer layer of microfibrils^1^,^11^,^17^,^18^ as it had been described based on electron microscopic analyses. The beaded structural features were also observed in GSD microscopy (Fig. 4). Citrine-TE appeared as globular structure that bordered the microfibrillar network. This structural organization had been previously proposed as a part of a model for elastic fibre formation from fibrillin-1-TE interaction studies^18^, where specific binding sites on the microfibrillar scaffold served as anchor for TE deposition. Furthermore, the microfibrils themselves appeared to be beaded in the GSD images. This could be accounted to the high resolution and an insufficient coverage with primary antibody used in GSD microscopy. However, detailed structural analysis of fibrillin-rich microfibrils suggested a periodicity within the tension free microfibril of approximately 56 nm, which is also reflected in the binding sites of the anti fibrillin-1 antibody 11C1.3^19^. As the same antibody has been used here to detect fibrillin-1, the beaded structure visible in GSD images could also depict this periodicity, as the resolution in GSD microscopy has been reported to range between approximately 20-30 nm for organic dyes^20^.

We used the confocal microscope equipped with temperature- and CO_2_-control to image Citrine-elastin transduced HDFneo over a period of several days. Figure 6 and the corresponding video demonstrate that elastogenesis is a temporally organized and dynamic process. Additionally, the growth curve shown in Supplementary Fig. S2 suggests that the speed in which elastic fibres are formed varies between different time points of cultivation. Especially it slows down towards the end as the amount of fibres seems to reach a plateau. Our model is therefor especially useful for the visualization and analysis of these dynamics in real-time, which is beyond routine immunocyto- and immunohistological protocols.

Here, we have presented an advanced human cell culture model using overexpression of Citrine-labelled human TE to generate functional fluorescence-labelled elastic fibres *in vitro*. We showed that overexpression of TE alone is sufficient to generate fibres, although TGF-β1 might stimulate further components that support elastin deposition. In super resolution imaging the model revealed a fibrillin-1 network on which elastin globules were deposited during elastogenesis and live cell imaging demonstrated the dynamics of a growing fibrous network over a period of several days. Taken together, we are confident that our model will help to further study and understand the process of elastic fibre formation in the human skin, which is particularly important when aiming for the design of regenerative medicine applications.

## Methods

### Cell Culture

HDFneo were purchased from tebu-bio (Offenbach, Germany). Cells were cultured in Dulbecco’s Modified Eagle Medium (DMEM) (Life Technologies, Carlsbad, USA) supplemented with 10% foetal calf serum (PAA Laboratories, Pasching, Austria), penicillin / streptomycin (Life Technologies, Carlsbad, USA), and L-glutamine (Life Technologies, Carlsbad, USA) (=full medium). Cells were maintained in a humidifying incubator at 37 °C and in a 5% CO_2_ atmosphere. Cell culture medium was changed every 3 to 4 days and cells were subcultured using trypsin-EDTA (PAA Laboratories) before reaching confluence. To stimulate elastogenesis, fibroblasts were seeded in appropriate culture dishes with a density of 20.000 cells/cm^2^ and treated with 10 ng/mL TGF-β1 (PeproTech, Rocky Hill, USA) or left untreated as control for up to 14 days.

### Lentiviral Fluorescent Citrine Tag

A human Citrine-TE construct was generated using the GeneArt gene synthesis and subcloning service of Life Technologies (http://www.lifetechnologies.com/GeneArt/). For human TE, a sequence was chosen that was published by Zhang *et al*.^21^ taken from human fibroblasts. This sequence was supplemented with six additional amino acids of exon 24 A as published by Fazio *et al*.^22^ also based on TE cDNA from human fibroblasts. For the fluorescence labelling of TE, a sequence for Citrine^23^ and a short linker sequence was chosen. Appropriate attB sites were added to the sequence to allow for cloning into a pDONR^TM^ Gateway® vector (Life Technologies, Carlsbad, USA). The whole sequence was synthesized by Life Technologies, cloned into a pDONR^TM^ Gateway® vector, and subsequently subcloned into a pLenti6/V5-DEST^TM^ Gateway® vector containing a CMV promoter, a C-terminal V5 epitope tag and a blasticidin resistance for selection in mammalian cells. The following production of lentiviral particles was also performed by Life Technologies.

Transduction of HDFneo with the Citrine-TE lentiviral particles was conducted by the following methods:

1. 10^5^ cells per well were seeded into a 6-well plate and incubated overnight. For transduction with the Citrine-TE lentiviral particles the medium was aspirated and replaced with antibiotic-free medium supplemented with 5 μg/mL polybrene (Millipore, Billerica, MA, USA) containing the desired amount of viral particles. After 24 h the virus-containing medium was removed and replaced with fresh full medium including antibiotics.
2. HDFneo were trypsinated, counted and resuspended in antibiotics-free medium (containing 5 μg/mL polybrene and the desired amount of viral particles) in a concentration of 5 × 10^5^ per mL. The suspension was then incubated in a water bath for 5 min at 37 °C and the cells subsequently seeded into cell culture flasks with additional polybrene-containing and antibiotics-free medium. 24 h later the medium was changed to full medium.

48 h after transduction, the medium was replaced with selection medium (full medium supplemented with 5 μg/mL blasticidin from Life Technologies, Carlsbad, USA) to select for successfully transduced cells using non-transduced cells as death control. Selected cells were subcultured and cryopreserved. Cells in passage two after transduction were thawed and used for experiments.

### Gene Expression Analysis

HDFneo were seeded into a 6-well plate with a density of 2 × 10^4^ cells/cm^2^ and stimulated with 10 ng/mL TGF-β1 (PeproTech) the following day or left untreated as a control. After 24 h, total RNA from cultured cells was isolated using the mirVana^TM^ isolation Kit (Life Technologies, Carlsbad, USA) following the manufacturers manual using 600 μL Lysis Buffer for one well of cells in a 6-well plate. Concentration of RNA was determined using a NanoDrop Spectrophotometer. Subsequently 1 μg of total RNA was then reverse transcribed into cDNA in 100 μL total volume using the High Capacity cDNA Reverse Transcription Kit (Life Technologies, Carlsbad, USA). mRNA levels were detected via quantitative Real Time TaqMan®–PCR using the 7900HT Fast-RT-PCR System (Applied Biosystems, Foster City, USA). FAM^TM^-labelled Primers (Applied Biosystems) were used for detection of elastin (Hs00355783_m1), fibrillin-1 (Hs00171191_m1), Citrine (Mr04097229_mr) and amounts of mRNA were normalized to 18 S rRNA (Hs99999901_s1) as a housekeeping gene. Relative gene expression was calculated using the 2^−ΔΔCT^ method^24^ with untreated cells as reference.

### IFA

As antibodies for elastin are (to our knowledge) not able to differentiate between the immature precursor TE and the mature cross-linked elastin, we refer to the antibody detectable protein as TE/elastin.

#### IFA of Fixed Cells

To analyse the ability of transduced and non-transduced HDFneo to generate elastic fibres, cells were seeded into chambered coverglasses (LabTek®, Nunc, Penfield, USA). Cells were grown for 14 days with 10 ng/mL TGF-β1 or left untreated as a control. Afterwards, cells were washed twice with PBS, fixed with methanol:acetone (1:1) at −20 °C and washed again twice with PBS at room temperature (RT). For immunostaining, samples were first incubated with PBS containing 1% BSA (blocking buffer) for at least 1 h at RT and subsequently labelled with the primary antibodies polyclonal rabbit anti human TE/elastin (1:200; Abcam) and monoclonal mouse anti fibrillin-1 clone 11C1.3 (1:100; Dianova GmbH, Hamburg, Germany) in blocking buffer. After three washing steps with PBS at RT, incubation with secondary antibodies was done with donkey anti rabbit Alexa 647 and donkey anti mouse Alexa 546 (both 1:1000; Life Technologies, Carlsbad, USA) in blocking buffer. Counterstaining of cell nuclei was carried out using DAPI (1 μg/mL in PBS; Roche, Basel, Switzerland).

#### IFA of Unfixed Cells

Transduced HDFneo were grown in chambered coverglasses (LabTek®, Nunc) under conditions specified in section cell culture with 10 ng/mL TGF-β1 before staining. The staining procedure was carried out at 4 °C with pre-cooled buffers. After washing the cells threefold with PBS, cells were incubated for 10 min with PBS containing 1% BSA (blocking buffer). Cells were labelled with primary antibody monoclonal mouse anti fibrillin-1 clone 11C1.3 (1:50; Dianova GmbH, Hamburg, Germany) in blocking buffer for 30 min followed by three rinses with blocking buffer. Samples were incubated with the secondary antibody donkey anti mouse KK943 (kind donation of the MPI Göttingen) or donkey anti mouse Alexa 647 (Life Technologies, Carlsbad, USA) respectively in blocking buffer for 30 min. Subsequently samples were washed twice with cold PBS and once with room temperature PBS before image acquisition with the confocal laser scanning microscope (CLSM) or the ground state depletion (GSD) microscope.

### Confocal Microscopy

A confocal laser scanning microscope, CLSM (Leica TCS SP5, Leica Microsystems, Mannheim), was used for image acquisition of fixed and immunostained elastic fibre models of transduced and non-transduced HDFneo. The instrument was used with a 20x objective (HCX PL APO CS 20.0 × 0.7 Dry UV, Leica) and with two hybrid detectors (HyD) and two photomultiplier tubes (PMT). Excitation laser wavelengths were 405 nm (DAPI), 488 nm (Citrine, EGFP), 561 nm (Alexa 546), and 633 nm (Alexa 647). Emission wavelength ranges were adjusted on the CLSM to 413–498 nm (DAPI), 499–571 nm (Citrine), 572–649 nm (Alexa 546), and 650–735 nm (Alexa 647). Single images of 1024 × 1024 pixels (775 × 775 μm) were generated with a bi-directional scanning rate of 200 Hz via a sequential scan to eliminate cross-talk. For maximum projections images were taken using a frame average of two frames per section taking 10 sections per vertical image stack. Step size between sections was 0.67 μm.

### Live Cell Imaging

A confocal laser scanning microscope (Leica TCS SP5, Leica Microsystems, Mannheim) was used for image acquisition of living Citrine-TE transduced HDFneo. The instrument was used with a 20x objective (HCX PL APO CS 20.0 × 0.7 Dry UV, Leica) and with two hybrid detectors (HyD). Excitation laser wavelength for Citrine was 488 nm and the emission wavelength range was adjusted to 500-610 nm. Additionally transmission light was detected to determine cell boarders. Z-Stacks (20 × 0.5 μm) with an xy-size of 1024 × 1024 pixels (775 × 775 μm) were generated with a bi-directional scanning rate of 200 Hz every hour using the autofocus function of the microscope software. The cells and the microscope were kept at 37 °C with a temperature control system (“The Cube & the Box”, Life Imaging Services, Reinach, CH). An active gas mixer (“The Brick”, Life Imaging Services, Reinach, CH) was used to provide the cells with a humidified environment and a constant 5% CO_2_-Level. The cells were imaged for 7 days in antibleaching medium DMEM^gfp^-2 (Evrogen, Moscow, RUS) supplemented with 10 ng/mL TGF-β1. Medium was changed on day 4 of image acquisition.

### Ground State Depletion Microscopy (GSD)

GSD microscopy is a super-resolution fluorescence microscopy method based on statistical switching of fluorophores between a dark “off”-state and a fluorescent ground “on”-state^15^. Using high laser intensities for excitation the ground state of a fluorophore can be depleted and the molecules are shifted into a dark “off” state with a comparable long lifetime. With a certain statistical probability a molecule leaves the dark state and returns to the ground “on”-state where it can run through several repetitive cycles of excitation and fluorescence emission before being shifted back to the dark state. As at a given time point only a small amount of molecules exhibit the “on”-state, excitation of these fluorophores results in punctual signals referring to one single molecule, from which the exact localisation of the molecule can be calculated. Recording a high number of frames (typically up to 100,000), localising the fluorophore in each detected event and summing up these localised signals results in a higher resolution than achievable in conventional light microscopy which is limited to half the wavelength of light.

For high-resolution image acquisition of unfixed and immunostained elastic fibre models of transduced HDFneo a GSD microscope was used (Leica SR GSD, Leica Microsystems, Mannheim, Germany) using the Leica Application Suite Advanced Fluorescence Software (version 3.2.0.9652, Leica Microsystems, Mannheim, Germany). The microscope is equipped with a 405 nm Laser (part of the Leica SR GSD System) and two continuous-wave lasers (MPB Communications, Montreal, Canada) of 488 nm and 642 nm; with an oil immersion objective lens (HCX PL APO 100x/1.47 oil, Leica) and an additional 1.6x lens creating an excitation spot of ~35 μm and with detection in epi-direction on a sCMOS PCO.edge camera (PCO AG, Kelheim, Germany).

The two colour-measurements of immunostained fibrillin-1 and Citrine were carried out separately in a buffer (GluOx/MEA) including TN buffer (50 mM Tris-HCl pH 8, 10 mM NaCl), 10% (w/v) glucose, 0.4 mg/mL glucose oxidase (Sigma-Aldrich) as oxygen scavenger, approx. 55 μg/mL catalase (Sigma-Aldrich) and 10 mM β-mercaptoethylamine (Sigma-Aldrich) (cf. Dempsey *et al*.^20^). KK943 (fibrillin-1) was recorded first using the 642 nm laser for depletion and acquisition; subsequently the 488 nm laser was used for depletion and acquisition of Citrine. Other image acquisition details are given in the corresponding captions. The obtained single super-resolution images were merged after acquisition to one dual-colour image.

### Image analysis

Image analysis of CLSM single mosaic images was performed using algorithms that are part of ImageJ, but were internally (at Beiersdorf AG, Hamburg, Germany) modified. On the base image we extracted the luminance channel by transforming the image to the L*a*b* colour space. We binarised the luminance intensities into background and foreground pixels where background only included pixels with intensities below 10. Using the ImageJ Particle Analyser, we calculated the fraction of the image that resembles the relative signal coverage. The adjusted algorithms were used to determine the relative signal coverage of a single mosaic image for each protein analysed (fibrillin-1, TE/elastin, Citrine) using the maximum projection of the single image. Single images out of z-range (e.g. if the nuclei were not imaged correctly) were left out of analysis. At least 3 of the 6 images per experimental condition could be used to generate a mean value of signal coverage. Acquisition settings were optimized for the TGF-β1 treated transduced HDFneo. This condition was used as a reference. Signal coverage for each other imaged protein within an experimental set-up was set in relation to this condition. The obtained relative values of three experimental set-ups were combined to an average value.

### Statistics

For statistical analysis the software OriginPro 8 G SR2 (Origin Lab Corporation, Northampton, MA, USA) was used. Significance levels were determined using the Mann-Whitney-Test and are given in the figure captions. Data are presented as Box Plots with the box giving percentiles (25,75), the horizontal line in the box being the median, the point in the box depicting the average and the whiskers showing outliers. The median value was chosen as it is more robust against outliers. For comparability averages and standard deviations are found online as Supplementary Tables S1 and S2.

## Additional Information

**How to cite this article**: Halm, M. *et al*. Visualizing tropoelastin in a long-term human elastic fibre cell culture model. *Sci. Rep.* **6**, 20378; doi: 10.1038/srep20378 (2016).

## Supplementary Material

Supplementary Information

Supplementary Information
