# Supplementary material for: Visualizing tropoelastin in a long-term human elastic fibre cell culture model
Source: Sci Rep. 2016 Feb 4;6:20378. doi: 10.1038/srep20378 (PMC4740895; doi:10.1038/srep20378)
Supplement: Supplementary Information [file srep20378-s2.pdf]

Visualizing tropoelastin in a long-term human elastic fibre cell culture model

M. Halm, K. Schenke-Layland, S. Jaspers, H. Wenck, F. Fischer

SUPPLEMENTARY INFORMATION

Supplementary Figures

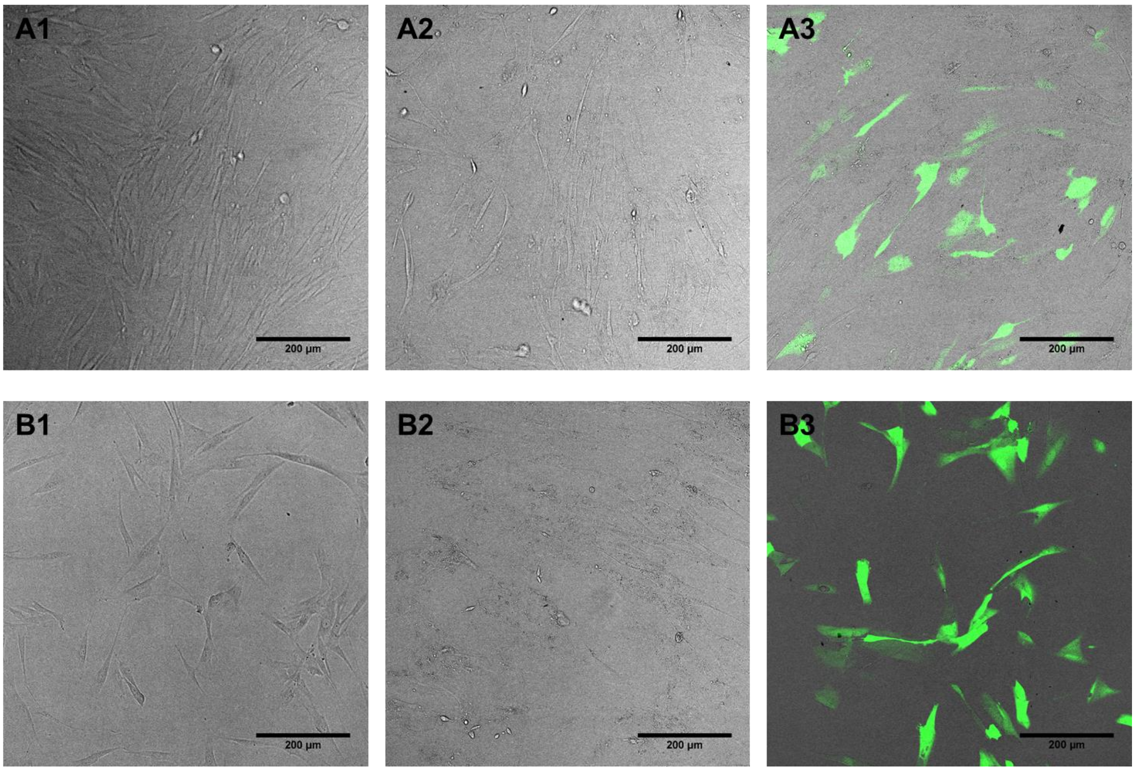

Supplementary Figure S1 | Confocal images of HDFneo transfected via lipofection (A) or electroporation (B). Images were acquired with a 20x dry objective on a Leica TCS SP5 microscope as described in "confocal microscopy" in the methods section of the main article. Excitation laser wavelength for EGFP was 488 nm and the emission range was set to 499-603 nm. Additionally the transmission was acquired to determine cell borders. **A** HDFneo were transfected with Lipofectamine® 2000 as described in supplementary methods and imaged 24 h after transfection. **B** HDFneo were transfected using the Neon® Transfection System as described in supplementary methods and imaged 24 h after transfection. **A1, B1** negative control (no DNA); **A2, B2** pEGFP-elastin; **A3, B3** positive control (pEGFP-C1).

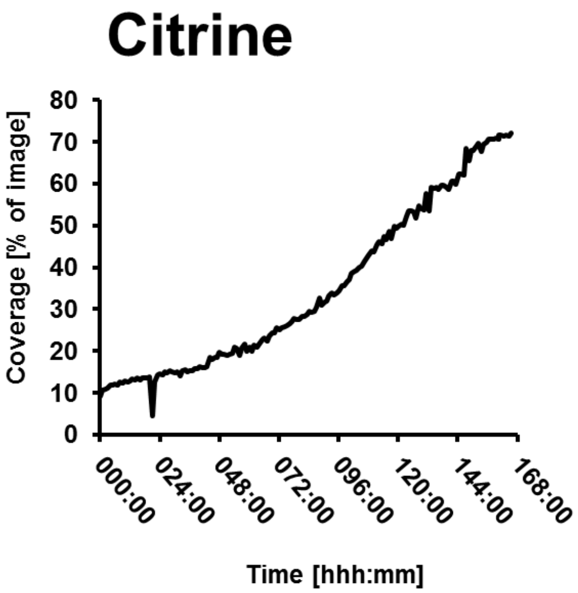

Supplementary Figure S2 | Growth curve of Citrine fluorescence observed live in Citrine-TE transduced HDFneo stimulated with 10 ng/mL TGF-β1. All single channel images representing Citrine from the time series shown in Fig. 6 and in the corresponding video in the Supplementary Information were analysed using the same method as described in "image analysis". Here, the received relative signal coverage for Citrine per image was plotted against the acquisition time which resulted in a growth curve of the developing Citrine-TE network.

Supplementary Tables

Supplementary Table S1 | Averages (Av) and standard deviations (SD) of the qRT-PCR data (fold changes) presented in Fig. 1 in the main article.

**Averages and standard deviations (Av ± SD)**	**elastin**	**fibrillin-1**	**Citrine**
Non-transd.	1.00 ± 0.00	1.00 ± 0.00	---
Non-transd. + TGF	2.63 ± 0.61	1.75 ± 0.13	---
Citrine-TE	7.53 ± 1.14	0.99 ± 0.09	1.00
Citrine-TE + TGF	10.24 ± 1.73	1.84 ± 0.33	1.06 ±0.13

Supplementary Table S2 | Averages (Av) and standard deviations (SD) of the image analysis data presented in Fig. 3 in the main article.

Averages and standard deviations (Av ± SD)	elastin	fibrillin-1	Citrine
Non-transd.	0.000 ± 0.000	0.759 ± 0.266	0.001 ± 0.000
Non-transd. + TGF	0.160 ± 0.155	0.820 ± 0.273	0.001 ± 0.001
Citrine-TE	1.154 ± 0.042	0.930 ± 0.205	0.199 ± 0.046
Citrine-TE + TGF	1.000 ± 0.000	1.000 ± 0.000	1.000 ± 0.000

Supplementary Materials & Methods

Plasmids. pEGFP-C1 (Clontech Laboratories, Mountain View, USA): mammalian expression vector carrying enhanced green fluorescent protein (EGFP), used as positive control; pEGFP-elastin (kind gift from the MPI Göttingen): mammalian expression vector carrying human elastin (see main methods section for sequence information) with a C-terminal EGFP tag.

Lipofection. HDFneo were seeded into 4-well Lab-Tek™ chambered coverglasses (50,000 cells/well) and incubated under standard conditions overnight in antibiotic-free medium. The following day the cells were transfected with the respective plasmid (or water for negative control) using Lipofectamine® 2000 (Life Technologies, Carlsbad, USA) according to the manufacturers manual. For each transfection sample 1 µg of plasmid DNA per 2 µL of transfection reagent was used. The following day cells were analysed for successful transfection under a Leica TCS SP5 microscope.

Electroporation. For electroporation the Neon® Transfection System (Life Technologies, Carlsbad, USA) was used with 100 µL tips according to the manual for adherent cells. Briefly, 3×10^5 HDFneo cells were mixed with 10 µg of plasmid DNA in 100 µL buffer R and treated with one pulse of 1700 V for 20 ms. Two of these 100 µL shots were combined with 4 mL antibiotic-free medium. From this suspension 500 µL per well were

seeded into 4-well Lab-Tek™ chambered coverglasses. The following day cells were analysed for successful transfection under a Leica TCS SP5 microscope.

Supplementary Notes

Transfection of HDFneo with pEGFP-elastin via lipofection and electroporation. In initial experiments we used lipofection and electroporation to express a fluorescence tagged elastin in HDFneo. Supplementary Fig. S1 shows images of non-transfected control cells (A1, B1), pEGFP-elastin transfected (A2, B2) and pEGFP-C1 transfected (A3, B3) cells. pEGFP-C1 was used as a positive control and is clearly expressed in HDFneo both after lipofection (A3) and after electroporation (B3) under the chosen conditions. Using the same conditions EGFP-tagged elastin could not be expressed in HDFneo, neither via lipofection (A2) nor electroporation (B2). The obtained images resemble those of the negative control (A1, B1) which clearly do not show any EGFP-specific fluorescence.
